# Predictive Value of Metabolic Parameters Derived From ^18^F-FDG PET/CT for Microsatellite Instability in Patients With Colorectal Carcinoma

**DOI:** 10.3389/fimmu.2021.724464

**Published:** 2021-08-26

**Authors:** Hao Liu, Zheng Ye, Ting Yang, Hongjun Xie, Ting Duan, Mou Li, Min Wu, Bin Song

**Affiliations:** ^1^Department of Radiology, West China Hospital, Sichuan University, Chengdu, China; ^2^Department of Nuclear Medicine, Sichuan Academy of Medical Sciences, Sichuan Provincial People’s Hospital, Chengdu, China

**Keywords:** PET/CT, metabolism, colorectal carcinoma, microsatellite instability, immunotherapy

## Abstract

**Background:**

Microsatellite instability (MSI) is one of the important factors that determine the effectiveness of immunotherapy in colorectal cancer (CRC) and serves as a prognostic biomarker for its clinical outcomes.

**Purpose:**

To investigate whether the metabolic parameters derived from^18^F-fluorodeoxyglucose (^18^F-FDG) positron emission tomography/computed tomography (PET/CT) can predict MSI status in patients with CRC.

**Materials and Methods:**

A retrospective analysis was performed on CRC patients who underwent ^18^F-FDG PET/CT examination before surgery between January 2015 and April 2021. The metabolic ^18^F-FDG PET/CT parameters of the primary CRC lesion were calculated and recorded with different thresholds, including the maximum, peak, and mean standardized uptake value (SUV_max_, SUV_peak_, and SUV_mean_), as well as the metabolic tumor volume (MTV) and the total lesion glycolysis (TLG). The status of MSI was determined by immunohistochemical assessment. The difference of quantitative parameters between MSI and microsatellite stability (MSS) groups was assessed, and the receiver operating characteristic (ROC) analyses with area under ROC curves (AUC) was used to evaluate the predictive performance of metabolic parameters.

**Results:**

A total of 44 patients (24 men and 20 women; mean ± standard deviation age: 71.1 ± 14.2 years) were included. There were 14 patients in the MSI group while there were 30 in the MSS group. MTV_30%_, MTV_40%_, MTV_50%_, and MTV_60%_, as well as TLG_50%_ and TLG_60%_ showed significant difference between two groups (all *p*-values <0.05), among which MTV_50%_ demonstrated the highest performance in the prediction of MSI, with an AUC of 0.805 [95% confidence interval (CI): 0.657–0.909], a sensitivity of 92.9% (95% CI: 0.661–0.998), and a specificity of 66.7% (95% CI: 0.472–0.827). Patients’ age and MTV_50%_ were significant predictive indicators of MSI in multivariate logistic regression.

**Conclusion:**

The metabolic parameters derived from^18^F-FDG PET/CT were able to preoperatively predict the MSI status in CRC, with MTV_50%_ demonstrating the highest predictive performance. PET/CT imaging could serve as a noninvasive tool in the guidance of immunotherapy and individualized treatment in CRC patients.

## Introduction

Colorectal cancer (CRC) is the third most common cancer and one of the major causes of cancer-associated mortality worldwide (1). Multiple treatments for primary and metastatic CRC have emerged, which includes curative surgery, radiotherapy, neoadjuvant chemotherapy, and immunotherapy (2). Immunotherapy is a treatment option that utilizes the body’s own immune system to attack cancer cells (3). Blocking immune checkpoints is currently one of the most promising approaches to activate therapeutic anti-tumor immunity and has shown encouraging results in CRC therapy (4).

However, the strong heterogeneity of CRC often leads to different prognosis and clinical outcome in patients who received similar treatment (5). As previously reported, chromosomal and genetic alterations that occur during the pathogenesis of CRC may be one of the contributing factors of heterogeneity (6). Microsatellites are short tandem repeat DNA sequences of one to three base pairs distributed throughout the human genome. Owing to their repeated structure, microsatellites are particularly prone to replication errors that are normally repaired by the mismatch repair (MMR) system. Microsatellite instability (MSI), caused by the absence of one or more MMR genes, has been considered as a reliable biomarker in the prediction of treatment response and prognosis in patients with CRC. Passardi et al. found that CRC with MSI selectively displayed highly upregulated expression of multiple immune checkpoints, including programmed death-1 (PD-1) and programmed death-ligand 1 (PD-L1) (7). In other words, CRC with MSI seem to be particularly responsive to immunotherapy, such as anti-PD-1 or anti-PD-L1 drugs. Moreover, MSI was identified as an important indicator in the selection of chemotherapy drugs of CRC (8). Recent studies showed that CRC with MSI was resistant to 5-fluorouracil (5-FU) chemotherapy and did not benefit from it (9). Typically speaking, MSI status is assessed with pathological specimen after surgery or biopsy, which is invasive and subject to sampling error (10). Therefore, a noninvasive method is urgently needed to preoperatively evaluate the MSI status and better facilitate the immunotherapy of CRC patients.

Medical imaging can capture information about the heterogeneity of the entire tumor noninvasively and could well predict tumor subtypes and the sensitivity of immunotherapy (11, 12). Moreover, imaging biomarkers were reported to be one of the effective indicators for predicting MSI expression in CRC (13–15). Wu et al. found that the quantitative imaging features derived from dual-energy computed tomography (DECT) showed good predictive performance for MSI status in CRC patients (13). In addition, radiomics-based artificial intelligence (AI), such as MRI-based deep learning models, also demonstrated optimal diagnostic capability for discriminating MSI from microsatellite stability (MSS) (14). However, the abovementioned studies either failed to reflect tumor metabolism or entailed complicated procedure, which was unavailable in clinical practice yet. Positron emission tomography/computed tomography (PET/CT) is a molecular imaging technique that can reflect tumor microenvironment and metabolic information with clinical radiotracer, such as ^18^F-fluorodeoxyglucose (^18^F-FDG) (16). A recent study showed that glucose metabolic response could predict anti-PD-1 therapeutic response in lung cancer (17). However, the role of metabolic parameters in CRC and MSI predictions remained unknown. Therefore, the purpose of this study was to investigate whether metabolic parameters derived from ^18^F-FDG PET/CT can predict MSI status in patients with CRC.

## Materials and Methods

### Patients

This retrospective study was approved by the Institutional Review Board of our institution, and the written informed consent was waived. We screened the medical records of patients who underwent preoperative ^18^F-FDG PET/CT followed by curative operations for CRC at our institution between January 2015 and April 2021. Detailed inclusion criteria were as follows: patients with (a) primary colorectal lesions diagnosed as colorectal adenocarcinoma by pathology; (b) complete preoperative whole body ^18^F-FDG PET/CT images before surgery within 3 months; (c) adequate immunohistochemical staining for the assessment of MMR protein expression.

**Figure 1:**
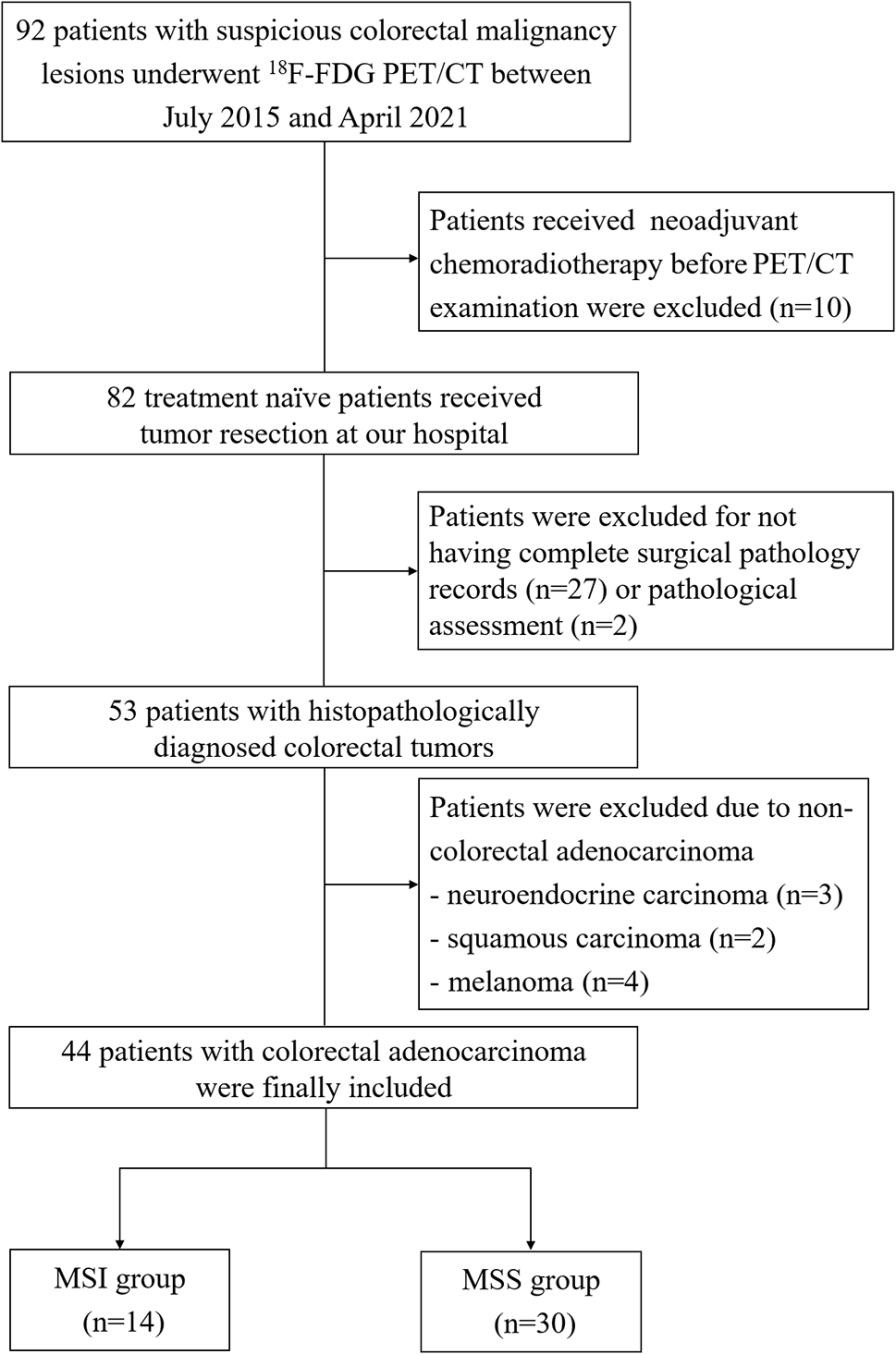
The flowchart of patient inclusion and exclusion.

We initially included 92 patients, and patients (a) who received neoadjuvant chemoradiotherapy (CRT) before^18^F-FDG PET/CT examination (*n* = 10); (b) without complete surgical pathology records (*n* = 27) or immunohistochemical assessment (*n* = 2); and (c) diagnosed as other pathological types (*n* = 9) were excluded (**Figure 1**).

### PET/CT Acquisition Protocol

All patients were asked to fast at least 8 h before the examination. In patients with diabetes, antihyperglycemic drugs were requested to stop for 12 h before the exam. The blood glucose of patients was tested before the injection of ^18^F-FDG, and the blood glucose level was required to be lower than 8 mmol/L. Patients were intravenously administered 5.5 MBq/kg ^18^F-FDG and received PET/CT examination after1 h rest.

PET/CT examination was performed with a hybrid scanner (Biograph Duo or Biograph mCTFlow64-4R, Siemens Healthcare Solutions Knoxville, TN). A non-contrast CT scan was firstly performed for localization and attenuation correction, with a slice thickness of 5 mm, a tube voltage of 120 kV, and tube current depending on the patient’s weight. Afterwards, PET images were acquired from the base of the skull to the proximal thigh for 3 min per bed position in a three-dimensional mode (Biograph Duo), or continuous table motion acquisitions (Biograph mCT Flow 64-4R). PET images were then reconstructed with an ordered method of ultra HD-PET, which included time of flight (TOF) and resolution recovery (TrueX) information.

### Image Analysis

All PET/CT images were analyzed by using Syngo TrueD VE40E workstation (Siemens Medical Solutions). The primary lesions were identified and analyzed by two experienced independent nuclear physicians who were blinded to any clinical and pathological information. Malignant lesions typically showed higher ^18^F-FDG concentration than that of the surrounding normal tissue. One of the physicians reviewed and analyzed all cases after a 2-week washout period.

**Figure 2:**
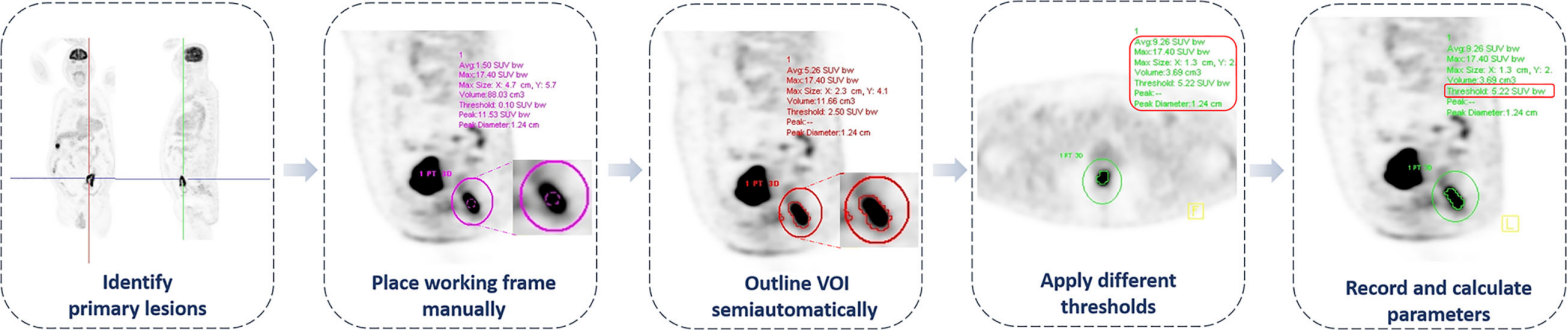
The workflow of ^18^F-FDG PET/CT metabolic parameter measurements.

**Figure 3:**
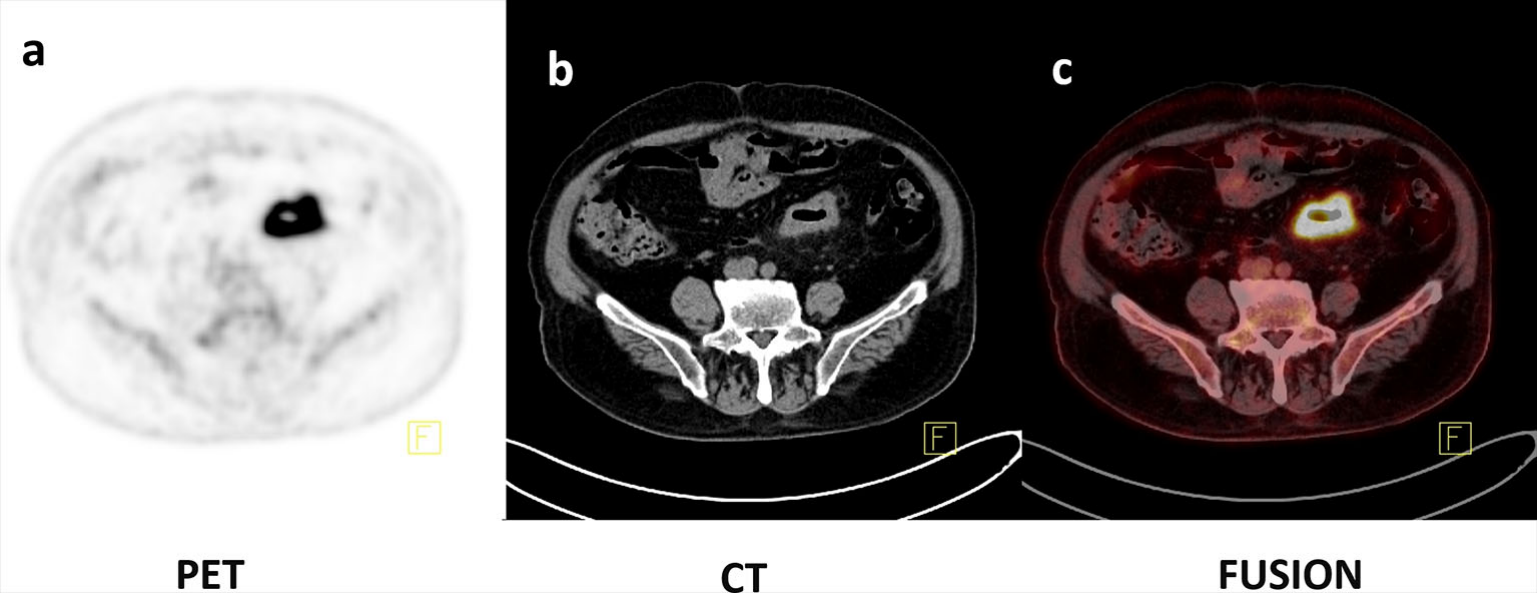
Axial PET, CT, and fusion images of an 86-year-old female with histopathologically proven CRC with MSI **(A–C)**. ^18^F-FDG PET/CT images showed that intense uptake in the sigmoid colon (SUV_max_ = 22.59, SUV_peak_ = 15.91, SUV_mean50%_ = 14.22, MTV_50%_ = 13.23 ml, TLG_50%_ = 188.13).

**Figure 4:**
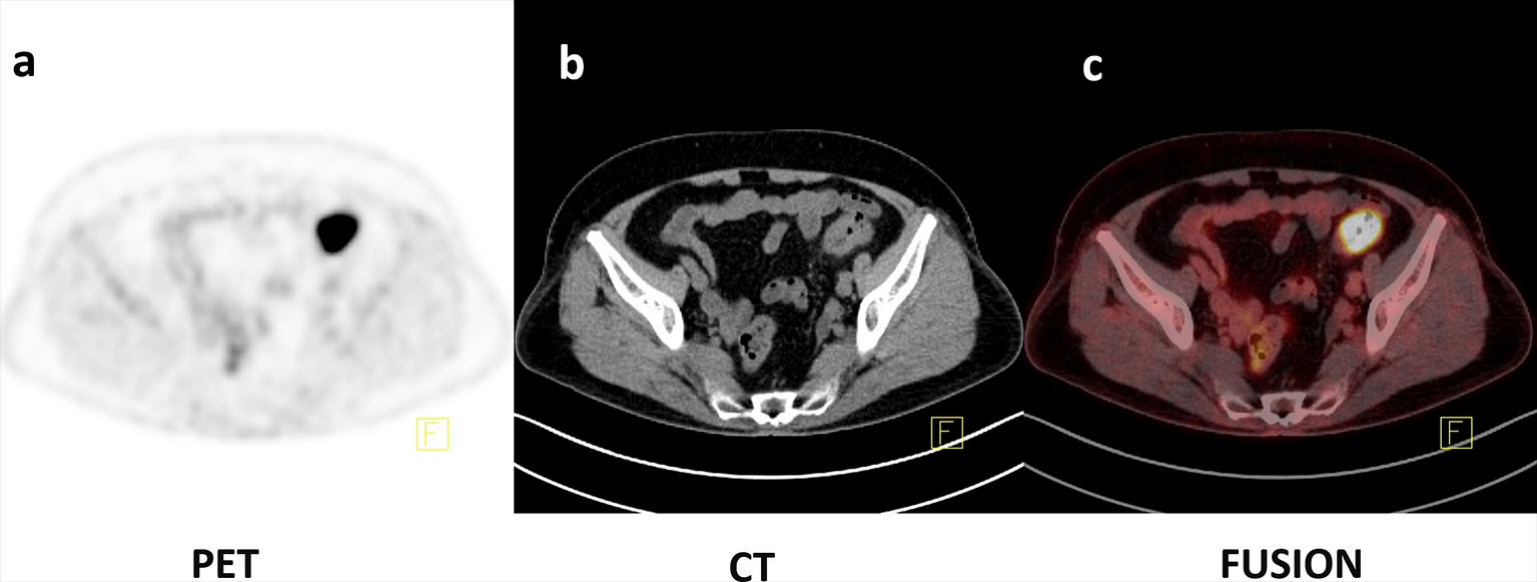
Axial PET, CT, and fusion images of a 77-year-old female with histopathologically proven CRC with MSS **(A–C)**. ^18^F-FDG PET/CT images showed that intense uptake in the sigmoid colon (SUV_max_ = 22.58, SUV_peak_ = 15.13, SUV_mean50%_ = 14.64, MTV_50%_ = 5.63 ml, TLG_50%_ = 82.4).

The characteristic of suspicious colorectal lesions was diagnosed according to the location, shape, size, radioactivity distribution, and standardized uptake value (SUV) of the lesions. The semi-automatic quantitative measurements started with a manually placed elliptical working frame, which was drawn large enough to include the primary lesion, and manual adjustment was performed to exclude adjacent lymph nodes, metastatic lesions, and high physiologic uptake organs (**Figure 2**). The SUV_max_ and SUV_peak_ would be automatically calculated and generated after the placement of the working frame. Afterwards, a three-dimensional volume of interest (VOI) would be automatically outlined with chosen threshold within the working frame. According to previous studies, two common methods were selected to set the threshold. The first one was using the fixed threshold, also known as fixed absolute threshold; the thresholds of 2.5, 3.0, 4.0, and 5.0 were applied (18, 19). The second approach was using the percent threshold, also known as fixed percentage threshold, setting the threshold between 30% and 60% with an increment of 10% of the SUV_max_ (20, 21). With different thresholds, metabolic tumor volume (MTV), and SUV_mean_ could be identified inside the segmented VOI. Additionally, total lesion glycolysis (TLG) was calculated by MTV multiplying SUV_mean_ (TLG = MTV × SUV_mean_), with representative images shown in **Figures 3**, **4**.

### Immunohistochemical Evaluation

All patients underwent surgical resection within 3 months of PET/CT examination. The conventional HE and immunohistochemical staining of resected specimens were evaluated by a senior pathologist in a blind fashion. Specifically, the general pathological types, differentiation grade, TNM stages, and the expression of MMR proteins (MLH1, PMS2, MSH2, and MSH6) were assessed.

Proficient mismatch repair (pMMR) was identified when all of the four proteins were intact and expected to be microsatellite stable (MSS). On the contrary, those with the loss of one or more abovementioned MMR proteins were referred to as defective mismatch repair (dMMR) and characterized as MSI.

### Statistical Analysis

The continuous variables were first checked for normality. The normally distributed data were expressed as the mean ± standard deviation (SD) and the difference was analyzed by Student’s *t* test, while the data were expressed as medians and interquartile ranges and tested by Mann–Whitney *U*-test if not normally distributed. The categorical variables were shown as the number of cases and percentages, and compared by chi-squared test (or Fisher’s exact test).

All quantitative PET/CT parameters were tested for inter-observer agreement and intra-observer agreement by interclass correlation coefficient (ICC) analysis, and ICC value greater than 0.90 was regarded as excellent agreement; between 0.75 and 0.90 as good agreement; between 0.5 and 0.75 as moderate agreement; and less than 0.50 as poor agreement. Receiver operating characteristic (ROC) analysis and the area under the ROC curve (AUC) were used to compare the predictive capabilities of quantitative PET/CT parameters between MSI and MSS group, and the sensitivity, specificity, optimal cutoff value, and 95% confidence interval (CI) were also calculated for each parameter. The comparison of different AUCs was conducted by the method described by DeLong et al. (22). Significant clinicopathological factors and the metabolic parameters with highest AUCs were entered into the multivariate logistic regression analysis.

All statistical analysis was performed by using SPSS software (version 22.0 IBM Corp, Armonk, NY) and MedCalc software (version 15.8). Two-sided *p*-values less than 0.05 were considered statistically significant.

## Results

### Patient Characteristics

A total of 44 patients were finally enrolled in this study (24 males, 20 females, age range 28–90 years, median age 73 years), with 14 patients in the MSI group and 30 patients in the MSS group. In the current cohort, patients in the MSI group were older and their tumors appeared more often in the colon than the MSS group. There was no difference in TNM stage, pathological general types, differentiation grade, and tumor size between two groups. Detailed patient information and clinicopathological characteristics are listed in **Table 1**.

**Table 1:** Clinicopathological characteristics of included patients.

Characteristics	Overall (*n* = 44)	MSI (*n* = 14)	MSS (*n* = 30)	*p*-value
Sex				
*Male*	24	6	18	0.342
* Female*	20	8	12	
Age (years)^a^	71.1 ± 14.2	81.4 ± 8.5	66.3 ± 13.8	0.001
Tumor size (cm)^b^	5 (3.6, 6)	5.25 (4.63,7.25)	4.5 (3.15,5.5)	0.351
Tumor location				
* Colon*	30	13	17	0.019
* Rectum*	14	1	13	
Pathological general types				
* Mass type*	10	2	8	0.462
* Ulcer type*	34	12	22	
Differentiation grade				
* Well or Moderate*	29	7	22	0.177
* Poor or Mucinous*	15	7	8	
AJCC-TNM stage				
* I–II*	16	7	9	0.313
* III–IV*	28	7	21	
T stage				
* T1–2*	5	1	4	1.000
* T3–4*	38	12	26	
N stage				
* Negative*	20	9	11	0.112
* Positive*	24	5	19	
M stage				
* Negative*	29	11	18	0.314
* Positive*	15	3	12	

a Data are presented in mean ± standard deviation (SD).

b data are presented in median (25th, 75th percentiles).

MSI, microsatellite instability; MSS, microsatellite stability; AJCC, American Joint Committee on Cancer.

### Inter-Observer and Intra-Observer Agreement of PET/CT Parameters

The excellent inter-observer and intra-observer agreement was found in SUV_max_ and SUV_peak_ with ICC values ranging from 0.999 to1.000. Other quantitative parameters also showed excellent inter-observer and intra-observer agreement with ICC values ranging from 0.958 to 0.999.

### Difference of PET/CT Parameters in MSI and MSS Groups

The detailed SUV_max_, SUV_peak_, SUV_mean_, MTV, and TLG in MSI and MSS groups are demonstrated in **Tables 2**, **3**. Specifically, MTVs were larger in CRC patients with MSI, with MTV_30%_, MTV_40%_, MTV_50%_, and MTV_60%_ showing significant difference (all *p*-values <0.05). Moreover, TLG_50%_ and TLG_60%_ were significantly larger in the MSI group than in the MSS group (all *p*-values <0.05). No significant difference was found in SUV_max_, SUV_peak_, and SUV_mean_ between two groups.

**Table 2:** SUV measurements of CRC patients.

	SUV_max_	SUV_peak_	SUV_mean_ with fixed threshold method				SUV_mean_ with percent threshold method			
			2.5	3.0	4.0	5.0	30%	40%	50%	60%
**MSI**	19.34 ± 8.67	14.80 ± 6.01	6.94 ± 1.90	7.61 ± 2.12	8.61 ± 2.27	9.35 ± 2.38	9.83 ± 4.0	11.08 ± 4.35	12.34 ± 4.90	13.72 ± 5.61
**MSS**	21.43 ± 10.14	16.03 ± 8.35	6.97 ± 2.02	7.55 ± 2.15	8.62 ± 2.31	9.54 ± 2.45	10.77 ± 5.09	12.34 ± 5.84	13.94 ± 6.63	15.56 ± 7.46
***p*-value**	0.509	0.821	0.965	0.929	0.986	0.806	0.549	0.476	0.427	0.417

MSI, microsatellite instability; MSS, microsatellite stability.

**Table 3:** MTV and TLG measurements of CRC patients.

	MTV with fixed threshold method				MTV with percent threshold method			
	2.5	3.0	4.0	5.0	30%	40%	50%	60%
**MSI**	72.0 (41.93,80.8)	62.97 (33.29,70.65)	50.92 (24.13,57.83)	41.60 (18.52,49.96)	38.24 (23.27,48.34)	27.38 (14.65,33.86)	17.40 (10.19,24.12)	10.16 (6.46,16.78)
**MSS**	40.21 (23.14,78.87)	34.12 (19.54,70.73)	25.83 (14.73,59.71)	20.37 (11.64,43.42)	17.90 (12.0,36.89)	11.79 (8.59,22.87)	7.49 (4.94,13.51)	4.45 (2.76,7.93)
***p*-value**	0.087	0.101	0.182	0.158	0.005	0.002	0.001	0.002
	**TLG with fixed threshold method**				**TLG with percent threshold method**			
	**2.5**	**3.0**	**4.0**	**5.0**	**30%**	**40%**	**50%**	**60%**
**MSI**	475.16 (209.33,632.36)	450.89 (209.33,632.36)	409.11 (178.06,582.65)	367.12 (156.62,546.46)	371.17 (185.63,474.80)	283.05 (137.02,413.55)	178.77 (106.44,335.98)	110.23 (69.57,228.48)
**MSS**	256.12 (136.95,600.94)	238.44 (129.04,578.47)	211.22 (114.0,528.22)	186.83 (100.83,476.36)	167.66 (105.18,167.66)	124.57 (86.95,316.87)	83.31 (68.57,223.38)	54.92 (43.47,105.0)
***p*-value**	0.208	0.226	0.208	0.208	0.091	0.07	0.028	0.022

MSI, microsatellite instability; MSS, microsatellite stability; MTV, metabolic tumor volume; TLG, total lesion glycolysis.

### Predictive Performance of PET/CT and Clinical Parameters for MSI

**Figure 5:**
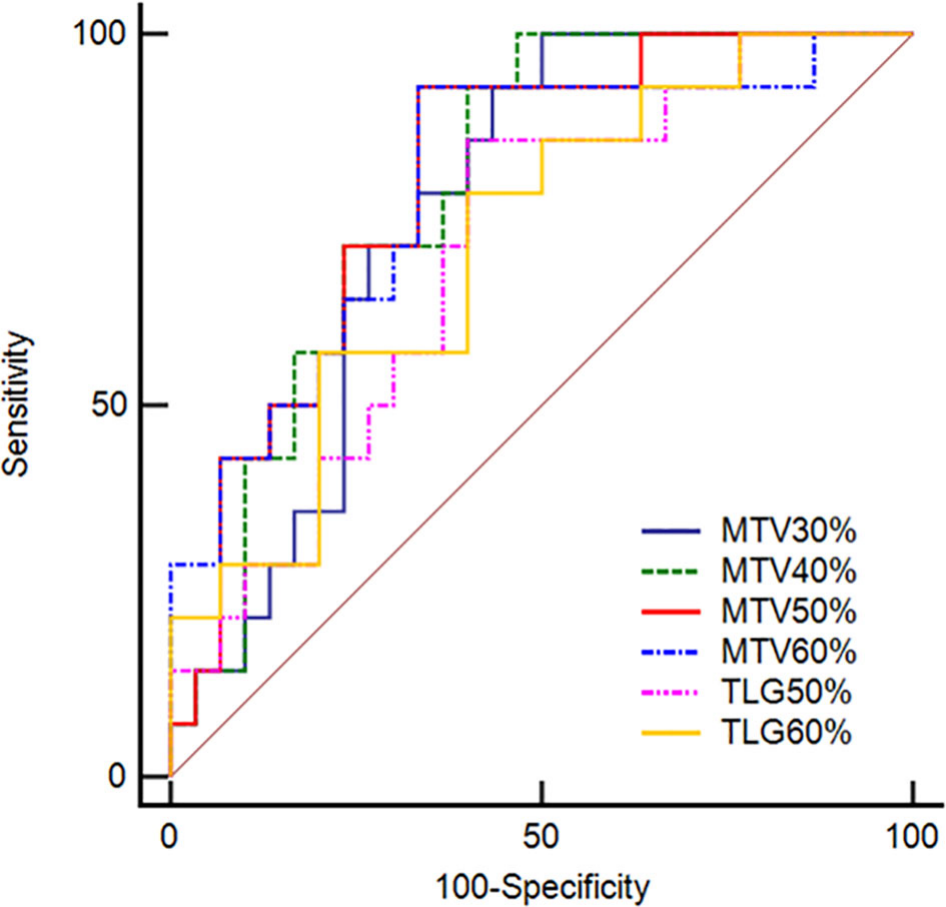
Receiver operating characteristic curves of MTV_30%_, MTV_40%_, MTV_50%_, and MTV_60%_ as well as TLG_50%_ and TLG_60%_ derived from PET/CT images for predicting MSI in CRC patients. The AUCs for predicting MSI from MSS were 0.764, 0.795, 0.805, and 0.795 for MTV_30%_, MTV_40%_, MTV_50%_, and MTV_60%_, respectively. In addition, the AUCs were 0.707 and 0.717 for TLG_50%_ and TLG_60%_.

For PET/CT parameters, MTV_50%_ demonstrated the highest predictive performance among the metabolic parameters, although not in a significant statistical way (all *p*-values >0.050),with an AUC of 0.805 (95% CI: 0.657–0.909), a sensitivity of 92.9% (95% CI: 0.661–0.998), and a specificity of 66.7% (95% CI: 0.472–0.827), respectively. The detailed predictive performance of PET/CT parameters and its ROC curves is illustrated in **Table 4** and **Figure 5**. For clinical parameters, the AUCs of age and tumor location were 0.843 (95% CI: 0.702–0.935) and 0.681 (95% CI: 0.523–0.813), respectively. No significant difference was found between AUCs of clinical and metabolic parameters (all *p* > 0.050). In multivariate logistic regression, age, tumor location, and metabolic parameters were entered. To avoid multicollinearity, we only selected one significant metabolic parameter in each category for assessment, which were MTV_50%_ and TLG_60%_. The odds ratio and *p*-value of age, tumor location, MTV_50%_, and TLG_60%_ were 0.819 (*p* = 0.005), 20.460 (*p* = 0.059), 0.831 (*p* = 0.039), and 1.008 (*p* = 0.276), respectively, which suggested age and MTV_50%_ as the independent predictive indicators for MSI.

**Table 4:** Predictive performance of metabolic parameters.

Parameters	AUC (95% CI)	Optimal cutoff value	Sensitivity (95% CI)	Specificity (95% CI)
**MTV _30%_**	0.764 (0.612,0.879)	19.27	0.857 (57.2–98.2)	0.600 (40.6–77.3)
**MTV _40%_**	0.795 (0.647,0.902)	13.25	0.929 (66.1–99.8)	0.600 (40.6–77.3)
**MTV _50%_**	0.805 (0.657,0.909)	8.89	0.929 (66.1–99.8)	0.667 (47.2–82.7)
**MTV _60%_**	0.795 (0.647,0.902)	5.62	0.929 (66.1–99.8)	0.667 (47.2–82.7)
**TLG _50%_**	0.707 (0.549,0.865)	97.02	0.857 (57.2–98.2)	0.600 (40.6–77.3)
**TLG _60%_**	0.717 (0.561,0.842)	74.11	0.786 (49.2–95.3)	0.600 (40.6–77.3)

AUC, area under the ROC curve; CI, confidence interval; MTV, metabolic tumor volume; TLG, total lesion glycolysis.

## Discussion

The current study revealed that age, tumor location, and certain metabolic parameters with specific thresholds had a significant difference between the MSI and MSS groups in patients with CRC. MTV and TLG with higher percentage thresholds showed good predictive ability in identifying MSI, especially theMTV_50%_.

With the advancement of image analysis technology, MTV and TLG, which are volumetric indexes derived from ^18^F-FDG PET, have been proposed for risk stratification of cancer patients (23). TLG is calculated by multiplying MTV by the SUV_mean_ of all voxels in the MTV, and represents both the degree of ^18^F-FDG uptake and the size of the tumor. In other words, MTV is affected by tumor size and the distribution of the SUV, and TLG is affected by the whole metabolic and volumetric burden of the tumor (24–26). In fact, MTV and TLG were considered to be more reliable markers reflecting tumor burden and aggressiveness, as these indexes provide tumor burden information and additional information that takes into account intra-tumoral biologic variation. In the current study, we found that MTV and TLG were significantly different between MSI and MSS groups, which was in agreement with the previous findings (27). Jiang et al. (27) retrospectively analyzed the pretreatment parameters of PET and reported the highest diagnostic performance of MTV_3.0_ and TLG_3.0_ in predicting PD-L1 expression level in CRC. On the contrary, SUV_max_, SUV_peak_, and SUV_mean_ did not show a significant difference between MSI and MSS groups in the current study. SUV_max_ and SUV_peak_ only represent certain parts of the tumor and reflect the maximum extent of glucose metabolism within tumor cells (28), while SUV_mean_ was the average of tumor glucose uptake value. Therefore, SUV itself provides limited information on tumor lesion, and heterogeneity might not be accurately shown by the SUV values.

The present study found that patients’ age and MTV_50%_ were significant predictive indicators of MSI, which was in agreement with a previous study (29) showing that CRC patients with MSI tended to be older. Similarly, Taieb et al. found that older age was associated with shorter survival after CRC recurrence (30). In addition, the predictive value of MTV with a higher percentage threshold tended to show better performance although not in a statistical way, which may due to the small sample size of the MSI group. Recent studies also confirmed that the different thresholds of MTV would affect the diagnostic performance. Bang’s study (21) found that MTV calculated using various thresholds was significantly associated with the 3-year disease-free survival (DFS) rate of locally advanced rectal cancer, and MTV calculated using a higher threshold (40%–70% SUV_max_) tended to be more strongly associated with 3-year DFS. In another study, various thresholds of metabolic parameters from FDG PET/CT were obtained and analyzed, and they found that TLG_40%_ can predict the treatment outcome of regorafenib in metastatic CRC (25). Moreover, we found that MTV with the percentage thresholds, rather than the fixed thresholds, showed better predictive performances of MSI status in patients with CRC. Previous studies also demonstrated percentage thresholds method as a better way of measurements, as it was an easily evaluated semiquantitative measurement of tumor textural heterogeneity and could minimize individual variations (19, 21). In addition, Henriksson et al. found the association between FDG uptake and the intra-tumoral heterogeneity in nude mice (31). On the other hand, Smedt et al. reported significant higher levels of cytotoxic T cells in the tumor and peritumoral region of MSI compared with MSS tumors (32). Therefore, MTV calculated using a higher threshold might be a representative marker for tumor clinicopathological characteristics in patients with CRC.

We acknowledged several limitations in our study. First, there may be selection bias due to the retrospective nature of this study. Second, the sample size was limited in this study, and further validation is required by including a large cohort. Finally, our results were obtained from a single institution, and further multicenter investigations are needed to validate the utility of these metabolic parameters for predicting MSI in patients with CRC.

In conclusion, the metabolic parameters derived from^18^F-FDG PET/CT were able to preoperatively predict the MSI status in CRC, with MTV_50%_ demonstrating the highest predictive performance. Using these parameters, the noninvasive evaluation of MSI can be achieved, and thus better guiding immunotherapy in CRC patients.

## Data Availability Statement

The raw data supporting the conclusions of this article will be made available by the authors, without undue reservation.

## Ethics Statement

The studies involving human participants were reviewed and approved by West China Hospital, Sichuan University. Written informed consent for participation was not required for this study in accordance with the national legislation, and the institutional requirements.

## Author Contributions

HL, TY, and BS contributed to conception and design of the study. HL and HX organized the image database and performed quantitative measurements. ZY, TD and ML performed the statistical analysis. HL and TY wrote the first draft of the manuscript. ZY, MW and BS revised the manuscript. All authors contributed to manuscript revision and read and approved the submitted version.

## Conflict of Interest

The authors declare that the research was conducted in the absence of any commercial or financial relationships that could be construed as a potential conflict of interest.

## Publisher’s Note

All claims expressed in this article are solely those of the authors and do not necessarily represent those of their affiliated organizations, or those of the publisher, the editors and the reviewers. Any product that may be evaluated in this article, or claim that may be made by its manufacturer, is not guaranteed or endorsed by the publisher.
